# Identification of a QTL region for ashy stem blight resistance using genome-wide association and linage analysis in common bean recombinant inbred lines derived from BAT 477 and NY6020-4

**DOI:** 10.3389/fpls.2022.1019263

**Published:** 2022-11-21

**Authors:** Diego M. Viteri, Angela M. Linares, Zoralys Miranda, Ainong Shi

**Affiliations:** ^1^ Department of Agro-environmental Sciences, University of Puerto Rico, Isabela Research Substation, Isabela, PR, United States; ^2^ Department of Agro-environmental Sciences, University of Puerto Rico, Lajas Research Substation, Lajas, PR, United States; ^3^ Department of Horticulture, University of Arkansas, Fayetteville, AR, United States

**Keywords:** ashy stem blight, common bean, *Macrophomina phaseolina*, *Phaseolus vulgaris*, quantitative trait loci, genome-wide association study

## Abstract

Ashy stem blight (ASB), caused by the fungus *Macrophomina phaseolina* (Tassi) Goidanich is an important disease of the common bean (*Phaseolus vulgaris* L.). It is important to identify quantitative trait loci (QTL) for ASB resistance and introgress into susceptible cultivars of the common bean. The objective of this research was to identify QTL and single nucleotide polymorphism (SNP) markers associated with ASB resistance in recombinant inbred lines (RIL) derived from a cross between BAT 477 and NY6020-4 common bean. One hundred and twenty-six F_6:7_ RIL were phenotyped for ASB in the greenhouse. Disease severity was scored on a scale of 1–9. Genotyping was performed using whole genome resequencing with 2x common bean genome size coverage, and over six million SNPs were obtained. After being filtered, 72,017 SNPs distributed on 11 chromosomes were used to conduct the genome-wide association study (GWAS) and QTL mapping. A novel QTL region of ~4.28 Mbp from 35,546,329 bp to 39,826,434 bp on chromosome Pv03 was identified for ASB resistance. The two SNPs, Chr03_39824257 and Chr03_39824268 located at 39,824,257 bp and 39,824,268 bp on Pv03, respectively, were identified as the strongest markers associated with ASB resistance. The gene Phvul.003G175900 (drought sensitive, WD repeat-containing protein 76) located at 39,822,021 – 39,824,655 bp on Pv03 was recognized as one candidate for ASB resistance in the RIL, and the gene contained the two SNP markers. QTL and SNP markers may be used to select plants and lines for ASB resistance through marker-assisted selection (MAS) in common bean breeding.

## Introduction

Ashy stem blight (ASB) is a common disease in the common bean (*Phaseolus vulgaris* L.) in tropical and subtropical regions in the Americas and worldwide (Kaur et al., 2012; Ambachew et al., 2021). The disease is caused by the seed-transmitted fungus *Macrophomina phaseolina* (Tassi) Goidanich, and the pathogen can infect the roots and all aerial plant parts during the entire cropping season (Islam et al., 2012; Kaur et al., 2012; Viteri and Linares, 2022a). Damping off, leaf burning, plant wilting, premature defoliation, and stem blight are the most common symptoms observed in infected plants (Kaur et al., 2012). Microsclerotia, which is the major fungal structure for the primary infection, can survive in the soil for more than 10 years (Short et al., 1980; Kaur et al., 2012), and different levels of aggressiveness between isolates have been reported (Miklas et al., 1998a; Mayek-Pérez et al., 2001a; Viteri and Linares, 2017). Yield losses up to 80% were reported in susceptible common bean cultivars (Mayek et al., 2003; Kaur et al., 2012; Viteri and Linares, 2022a).

Genetic resistance is a better strategy than crop rotation to combat ASB, and the use of fungicides is not adequate to control this disease efficiently (Singh and Schwartz, 2010). Low to high levels of resistance have been reported in common bean and tepary bean (*Phaseolus acutifolius* A. Gray). For instance, the common bean genotypes of BAT 477, IPA 1, ‘Negro Tacaná’, ‘Negro Perla’, ‘San Cristobal 83’, TARS-MST1, and XAN 176 (Pastor-Corrales and Abawi, 1988; Mayek-Pérez et al., 2001b) and tepary bean accessions of Mex-114, PI 440806, and PI 321637 (Miklas et al., 1998a) were reported with higher levels of ASB resistance in field evaluations. Conversely, Andean common bean genotypes A 195, ‘Badillo’, ‘PC 50’, and PRA154 were reported in previous studies as having partial resistance in greenhouse evaluations (Viteri and Linares, 2017; Viteri et al., 2019). However, some breeding lines (e.g., BAT 477, NY6020-4, XAN 176) can have susceptible scores at later reproductive stages by the cut-stem method and two inoculations of *M. phaseolina* (Viteri et al., 2019). Furthermore, avoidance mechanisms (e.g., plants with upright growth habits) can help to reduce disease severity in the field and prevent a susceptible response of some genotypes (Mayek-Pérez et al., 2001a; Mayek-Pérez et al., 2001b; Viteri and Linares, 2022a).

ASB resistance can be inherited qualitatively or quantitatively depending on the resistant host genetic background and is affected by the screening method and environment used. For example, two complementary dominant genes (*Mp*-*1* and *Mp*-*2*) were identified to confer resistance in BAT 477/A 70 F_2_ population screened in growth chambers (Olaya et al., 1996). Likewise, Mayek-Pérez et al. (2009) reported that two dominant genes with double recessive epistasis and nine quantitative trait loci (QTL) derived from BAT 477 were involved in field resistance to *M. phaseolina*. In addition, nine QTL on chromosomes Pv03, Pv05, Pv06, Pv08, Pv09, and Pv10 were reported to confer ASB resistance in the field and controlled environments in recombinant inbred line (RIL) population derived from BAT 477 and UI-114 (Méndez-Aguilar et al., 2017). Furthermore, Miklas et al. (1998b) reported that five QTL on Pv04, Pv06, Pv07, and Pv08 provided field resistance to ASB in the Dorado/XAN 176 RIL population, and they were derived from the black common bean XAN 176. More recently, Viteri and Linares (2019) identified two recessive genes and one recessive gene conferring resistance to *M. phaseolina* in PC 50/’Othello’ and ‘Badillo’/PR1144-5, respectively, under greenhouse conditions. These genes were derived from Andean genotypes PC 50 and Badillo. In the same study, one dominant gene was involved in resistance in the A 195/PC 50 population. To the best of our knowledge, the molecular identification of resistant QTL to ASB involving crosses between Andean and American genotypes has not been reported. This would be useful in marker assisted selection to increase the levels of resistance in common bean cultivars. The objective of this research was to identify QTL and SNP markers associated with ASB resistance in common bean RIL derived from a cross between BAT 477 and NY6020-4 genotypes. This would be useful in studying ASB resistance in different common bean genetic backgrounds, and the associated SNP markers could be used to select ASB resistant plants and lines in common bean molecular breeding through marker-assisted selection (MAS).

## Materials and methods

### Plant material and RIL development

A cross between BAT 477 and NY6020-4 common bean lines was made at the Isabela Research Substation at the University of Puerto Rico in January 2017. One hundred and twenty-six F_6:7_ RIL from BAT 477/NY6020-4 was developed by single-seed-descent method from the F_2_. NY6020-4 is an Andean snap bean with a determinate growth habit (Viteri et al., 2015) and low to partial levels of resistance to ASB (Viteri and Linares, 2017). BAT 477 is a common breeding line with indeterminate prostrate growth habit type III (Singh, 1982). This genotype was reported to be tolerant to drought stress (Arruda et al., 2018), and it has been widely used as a source of resistance to ASB. However, low to high levels of resistance were reported in previous studies in the greenhouse and field (Mayek-Pérez et al., 2009; Viteri and Linares, 2017; Viteri and Linares, 2022a; Viteri et al., 2019). BAT 477 was selected in this study because of the importance of identifying resistant QTL to the direct exposure of the pathogen, and to avoid a confounded effect of QTL expressed in field evaluations that could be associated with drought and heat stresses and/or disease avoidance mechanisms. NY6020-4 was selected because white beans are the most important market class in Puerto Rico (Beaver et al., 2020).

### *Macrophomina phaseolina* isolates

PRI19 and PRL19 *M. phaseolina* isolates were collected from an infected stem tissue of common bean at R5 stage in the field of the Research Substations in Isabela (February, 2019) and Lajas (May, 2019), respectively. The fungi were isolated from infected stem tissue at reproductive stages (R5) with the characterized stem blight symptom. In addition, PRI21 was isolated from an infected seedling planted in the greenhouse in Isabela in January 2021. These three isolates were used in this study.

### Phenotyping of ashy stem blight resistance

The 126 RIL and their parent strains were screened for resistance to PRI19 *M. phaseolina* isolate in Isabela and PRL19 in Lajas, respectively, in September 2020; they were screened for resistance to PRI21 isolate in Isabela in February 2021. A randomized complete block design (RCBD) with three replications were used, and four plants of each RIL line per replication were planted in each experiment in greenhouse trials.

One inoculation per plant of each of the aforementioned *M. phaseolina* isolates was conducted at the fourth internode (V5 growth stage). A 200 μL Eppendorf tip stacked with four mycelial plugs from a 48-hour-old *M. phaseolina* culture growth at 28°C on potato dextrose agar was used for each inoculation. Inoculated plants were exposed to high mean day temperatures > 27°C, and moisture ranged from 50–70%, which promoted an adequate ASB infection (Pastor-Corrales and Abawi, 1988; Mayek-Pérez et al., 2002; Viteri and Linares, 2022a). The disease severity was evaluated at 42 d after inoculation. A 1–9 scale was used, where 1 signified no sign of pathogen infection, 3 signified that the fungus did not pass the first node above/below the point of the inoculation, 6 signified that *M. phaseolina* reached the second node above/below the point of the inoculation, and 9 signified that the pathogen passed the third node below the point of inoculation with or without plant death (Singh et al., 2014; Viteri and Linares, 2017). Plants with scores of 1–3 were considered resistant, 4–6 intermediate, and 7–9 susceptible (Viteri and Linares, 2017).

### Phenotypic data analysis

Disease scores of ASB phenotypic data were analyzed by analysis of variance (ANOVA) using the general linear model procedure of JMP Genomics 9 (SAS Institute, 2012 Cary, NC). The descriptive statistics were generated using ‘Tabulate’; the distribution of the data was drawn using ‘Distribution’; and Pearson’s correlation coefficients (r) were calculated using “Multivariate Methods” of JMP Genomics 9 (SAS Institute, 2012 Cary, NC). The least squares mean of each isolate resistance for each RIL line was used as the phenotypic data for GWAS and QTL mapping using the ANOVA method.

Broad-sense heritability (H) was estimated using the following formula (Holland, 2003)


$$\text{H}=\sigma_{\text{ G}}^{2}/\left[\sigma_{\text{ G}}^{2}+(\sigma_{\text{ GE}}^{2}/\text{e})+(\sigma_{\text{ E}}^{2}/\text{re})\right]$$

where $\sigma_{\text{ G}}^{2}$ is the total genetic variance; $\sigma_{\text{ GE}}^{2}$ is variance between genetic and block interaction; $\sigma_{\text{ E}}^{2}$ is the residual variance; e is the number of environments; and r is the number of replications. The estimates for $\sigma_{\text{ G}}^{2}$ , $\sigma_{\text{ GE}}^{2}$ and $\sigma_{\text{ E}}^{2}$ are $\sigma_{\text{ E}}^{2}$ = MS_E_; $\sigma_{\text{ GE}}^{2}$ = (MS_GE_ – MS_E_)/r; and $\sigma_{\text{ G}}^{2}$ = (MS_G_ – MS_GE_)/re.

Phenotypic data of each of the three *M. phaseolina* isolates, PRI19, PRL19, and PRI21, were analyzed, separately. Because PRI19 and PRI21 were collected from the same location of Isabela, Puerto Rico, we merged the ASB phenotypic data as PRI. We also merged the ASB phenotypic data of the three isolates as PRI.L. Therefore, five ABS data sets performed GWAS and QTL mapping for ABS resistance in this study.

### DNA extraction, sequencing, and SNP genotyping

Genomic DNA was extracted using the DNeasy^®^ plant mini kit (Qiagen, Germantown, MD). The DNA was extracted from a bulk sample of emerging trifoliate leaves collected from three plants of each parent and the 126 RIL. The DNA concentration was adjusted to 10 ug/mL using a Nanophotometer^®^ P-class (Implen, Westlake Village, CA). Whole-genome resequencing (WGR) with 2x common bean genome size coverage took place on the 128 samples (126 RIL plus two parents) in Texas A&M Genomics and Bioinformatics Center. Libraries were prepared with PerkinElmer NEXTFLEX Rapid XP kit protocol, and common bean samples were sequenced on Illumina NovaSeq S4 XP using the 2x150 bp recipe. FASTQ files were processed with the Illumina Dynamic Read Analysis for Genomics (DRAGEN) Bio-IT processor. The DRAGEN pipeline (v3.8.4) was used to obtain SNP data for each individual sample based on the genome reference of *P. vulgaris* v2.1 common bean genome and annotation (https://phytozome-next.jgi.doe.gov/info/Pvulgaris_v2_1).

A total of 6,463,014 SNPs were identified in the 126 RIL and their parents, distributed on the 11 chromosomes. In the RIL population, the relevant SNP should contain two homozygous alleles in a 1:1 ratio with each other. A chi-square test was performed for each of the 6,463,014 SNPs found in DNA sequencing. We retained SNPs that had two homozygous alleles in a 1:1 ratio, those with a chi-square test P-value > 0.01, and the two parents which had different alleles and homogeneity. Meanwhile, we also filtered each SNP and kept the SNPs with missing alleles < 5%, heterogeneous rate < 5%, and minor allele frequency (MAF) > 35%. After filtering, the retained 72,017 SNPs distributed on 11 chromosomes were used in this study (**Supplementary Figure S1**). The 72,017 SNPs across the 126 RIL and their two parents (BAT 477 and NY6020-4) have been published at https://doi.org/10.6084/m9.figshare.19919221.v1.

### Association analysis

GWAS was performed using the 72,017 SNPs across the 126 RIL by SMR (single marker regression), GLM (general linear model), and MLM (mixed linear model) methods in TASSEL 5 (Bradbury et al., 2007), and by GLM, FarmCPU (fixed and random model circulating probability unification), and BLINK (Bayesian-information and linkage-disequilibrium iteratively nested keyway) models in GAPIT 3 (Genomic Association and Prediction Integrated Tool version 3) (Wang and Zhang, 2021; https://zzlab.net/GAPIT/index.html; https://github.com/jiabowang/GAPIT3) by setting PCA = 2. In addition, a *t*-test was conducted for all 72,017 SNPs by using visual basic codes in Microsoft Excel 2016.

Multiple TASSEL and GAPIT models were used to find reliable and stable ASB resistance-associated SNP markers and candidate genes and QTL regions in the RIL. The significant threshold of associations was calculated using Bonferroni correction of P-value with an α = 0.05 (0.05/SNP number) as the significance threshold (López-Hernández and Cortés, 2019), and LOD value of 6.16 was used as significance threshold based on the 72,017 SNPs in this study. In addition, a *t*-test was conducted for all 72,017 SNPs by using Visual Basic codes in Microsoft Excel 2016.

### Genetic mapping and QTL analysis

Linkage maps were constructed for the RIL population using JoinMap 4 (Van Ooijen, 2006) and MSTmap (Wu et al., 2008; http://mstmap.org/). Single marker regression (SMR), single-trait multiple interval mapping (SMIM), and single-trait CIM MLE (SMLE, single-trait composite interval mapping maximum likelihood estimation) analyses were conducted for QTL mapping using QGene (Joehanes and Nelson, 2008).

### Candidate gene identification/detection

Genes were searched within the QTL region from the *P. vulgaris* genome reference version v2.1 (https://phytozome-next.jgi.doe.gov/info/Pvulgaris_v2_1). Our objective was to find analogs of disease resistant genes near the significantly associated SNP markers in the QTL region for ASB resistance.

## Results

### Ashy blight resistance in the RIL

**Figure 1:**
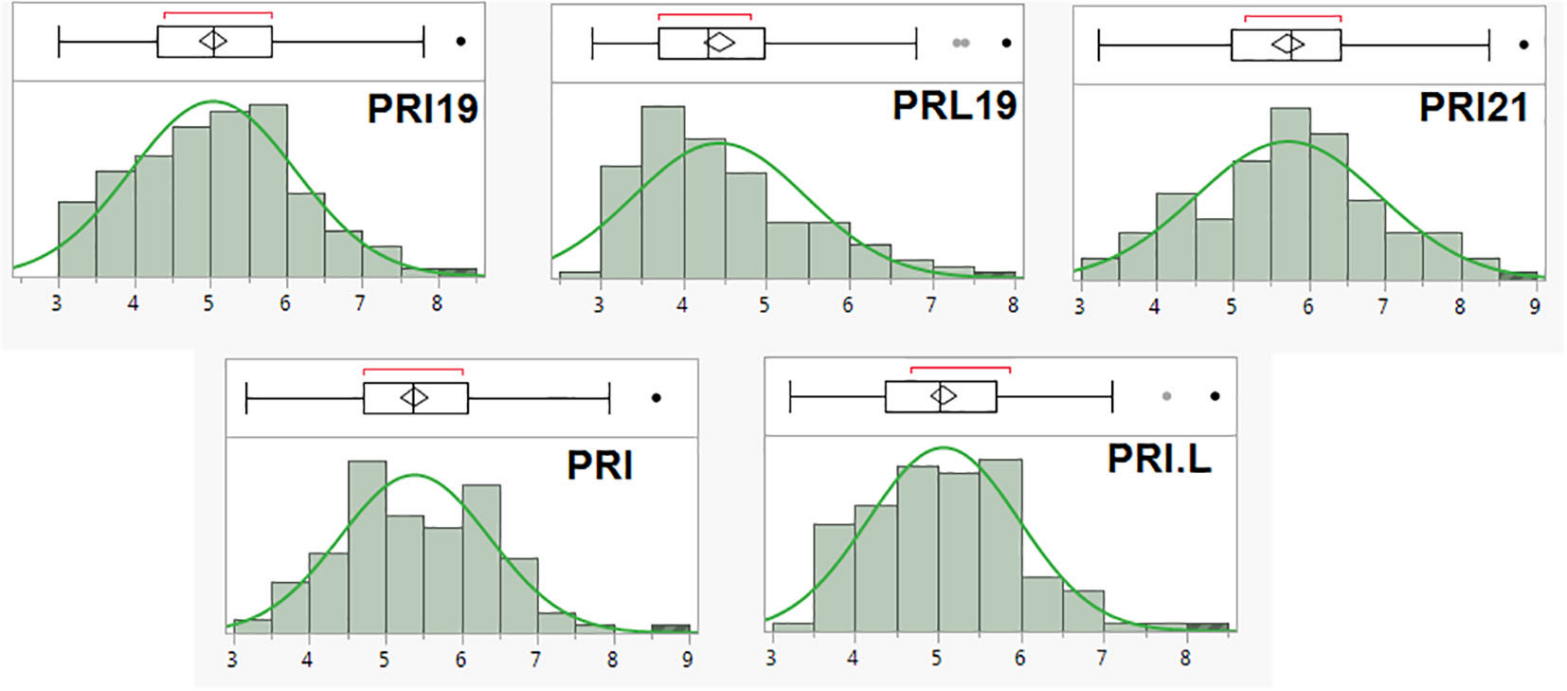
The distribution of ashy stem blight (ASB) score (1-9 scale) in 126 common bean RIL of BAT 477 and NY6020-4 for resistance to five *Macrophomina phaseolina* isolates or combinates, where the x axis represents ASB score (1-9 scale) and the y axis represents number of RIL.

The scale (1–9) of ashy blight resistance in the 126 RIL derived from BAT 477 and NY6020-4 showed a near normal distribution in all five pathogen combinations (**Figure 1**). The mean disease rate ranged from 3.0–8.3, 2.9–7.9, 3.2–8.8, 3.2–8.6, and 3.2–8.4; averaged 5.0, 4.4, 5.7, 5.4, and 5.1 with a standard deviation of 1.09, 1.05, 1.21, 0.99, and 0.93, and the coefficient of variation (CV) was 21.6%, 23.6%, 21.1%, 18.4%, and 18.0%, for PRI19, PRL19, PRI21, PRI, and PRI.L, respectively (**Supplementary Table S1**). The data showed an extensive range and variation of the ASB disease scale in the 126 RIL, confirming the suitability of the RIL for GWAS and QTL analyses.

Broad-sense heritability was 46.3%, 63.5%, 53.2%, 71.2%, and 68.7% for PRI19, PRL19, PRI21, PRI, and PRI.L, respectively (**Table S2**), indicating the ASB resistance was mediate highly inheritable.

There were strong correlations (r = 0.36–0.98), where 5 of the 10 r values were greater than 0.80, and 8 out of 10 were greater than 0.60 of ASB resistance scores among the five pathogen combinations in the 126 RIL (**Table S3**), suggesting that the combinations had similar genetic resistance.

### Association study

**Figure 2:**
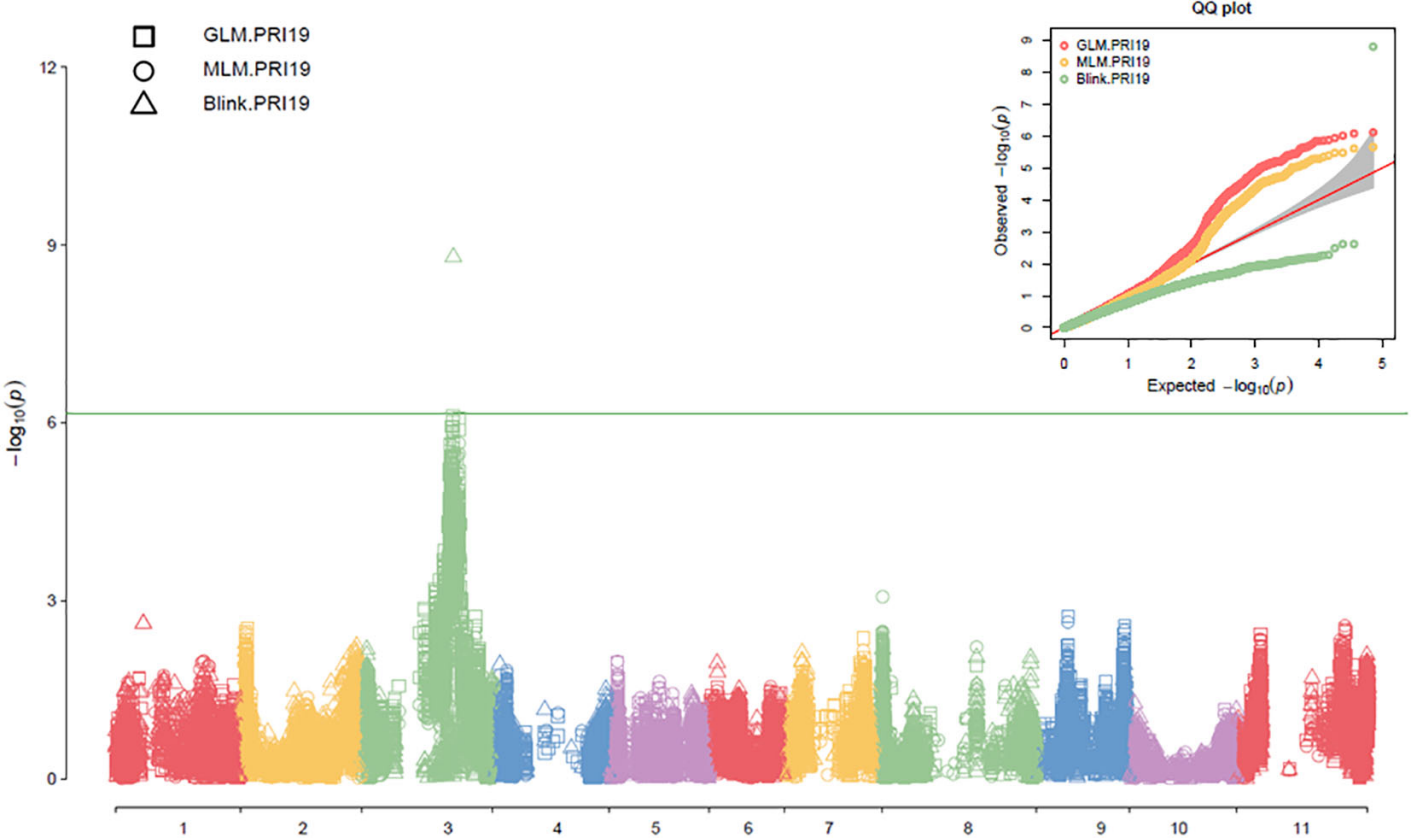
The multiple Manhattan and QQ plots of GLM, MLM, and BLINK models for ashy stem blight PRI 19 pathogen resistance in 126 common bean RIL of BAT 477 and NY6020-4.

**Figure 3:**
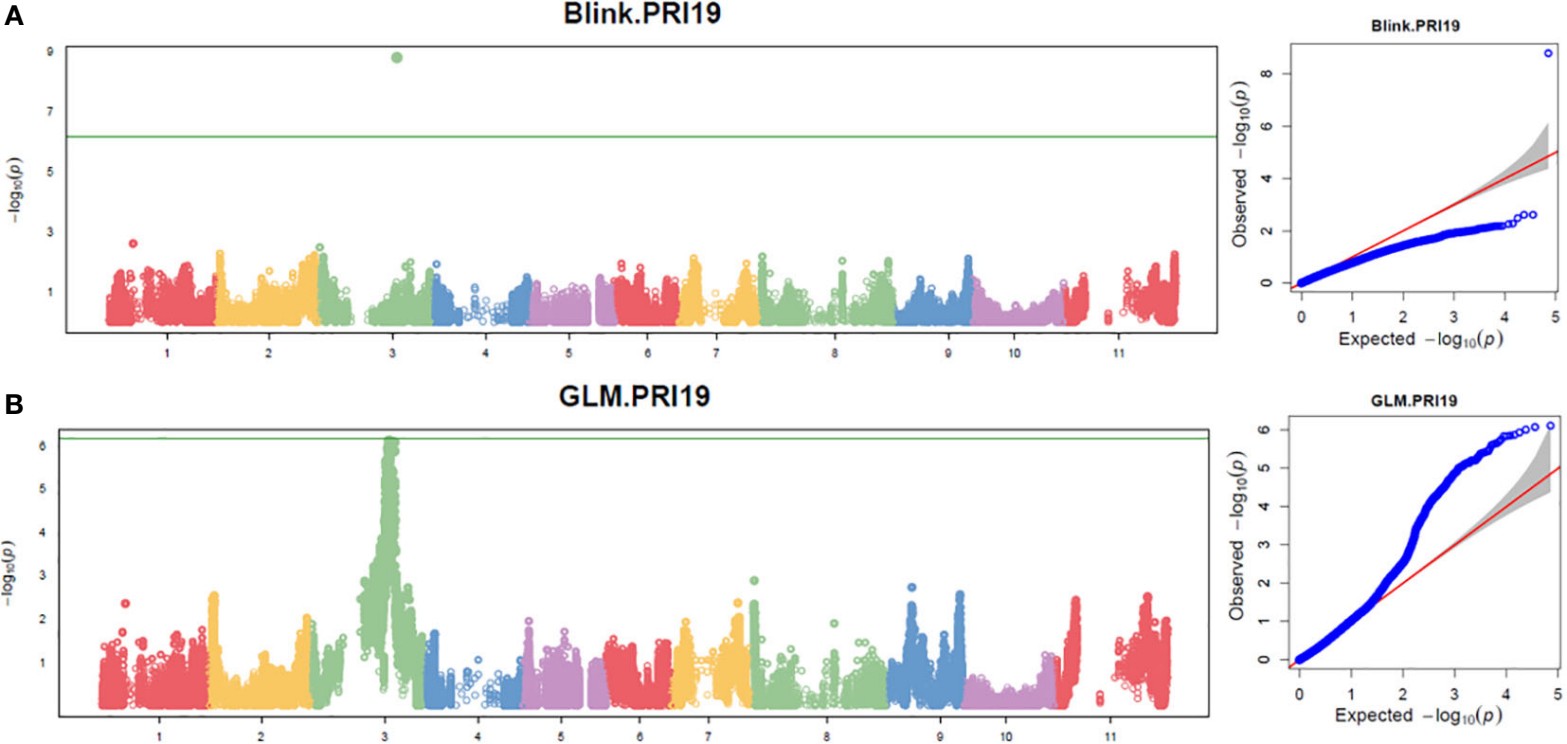
The Manhattan and QQ plots of Blink **(A)** and GLM **(B)** models for ashy stem blight PRI 19 pathogen resistance in 126 common bean RIL of BAT 477 and NY6020-4.

Three models, GLM, MLM, and Blink in GAPIT 3, and three models, SMR, GLM, and MLM in TASSEL 5 when PCA = 2 performed association analysis for ASB resistance in this study. The observed vs expected LOD [-log_10_(p)] distributions in QQ-plots showed a large divergence from the expected distribution based on multiple QQ plots based on three models (GLM, MLM, and Blink) in PRI19, PRL19, PRI21, PRI, and PRI.L (**Figure S2B** on right side), indicating there were SNPs associated with ASB resistance in the association panel. The multiple Manhattan plots on three models (GLM, MLM, and Blink) in PRI19, PRL19, PRI21, PRI, and PRI.L (**Figure S2A** on left side) showed that a dozen SNPs with LOD value greater than 6.16 (significant threshold) were associated with ASB resistance. The multiple Manhattan and QQ plots based on the three models for ASB PRI19 resistance are also shown in **Figure 2**. The QQ-plots and Manhattan plots of three models in Tassel 5 (**Figure S3** listed ASB PRI resistance) showed similar trends to GAPIT3 for ASB PRI resistance, indicating that there were significant SNP markers on Pv03 associated with ASB resistance. The Manhattan and QQ plots based on either Blink or GLM showed that there were SNPs on Pv03 associated with the ASB resistance for PRI19 (**Figure 3**), for PRL19 (**Figure S4**), for PRI21 (**Figure S5**), for PRI (**Figure S6**), and for PRI.L (**Figure S7**), further validated by QTL on Pv03 for ASB resistance.

Based on the three models in GAPIT 3 and the three models in TASSEL 5 when PCA = 2, 45 SNPs, located in the region of ~4.28 Mbp from 35,546,329 bp to 39,826,434 bp on chr 3, were associated with the ASB resistance with an LOD [-log_10_(p)] > 6.16 in one or more of the six models for one or more pathogen combination (**Table S4**; **Figure S2**). *t*-test showed all SNPs had an LOD > 2.0 accept Chr03_3572932 for PRI21 resistance (**Table S4**), validating 45 SNPs associated with ASB resistance at P=0.01 level. The averaged LOD ranged from 2.74 to 4.78 based on the six GWAS models and 3.52 to 6.12 based on *t*-test, and the R-square was 11.2 – 17.7% averaged from the six models (**Table S4**), indicating that there is a QTL on Pv03 for ASB resistance.

After combined analysis of the six GWAS models, four SNPs, Chr03_37381665, Chr03_37616128, Chr03_39824257, and Chr03_39824268 were associated with PRI19 resistance; three SNPs, Chr03_38912965, Chr03_38926573, and Chr03_39009342 with PRI21 resistance; four SNPs, Chr03_35546329, Chr03_35847673, Chr03_36036641, and Chr03_36036679 with PRL19; four SNPs, Chr03_38912965, Chr03_39009342, Chr03_39824257, and Chr03_39824268 with PRI; and five SNPs, Chr03_37616128, Chr03_38912965, Chr03_39009342, Chr03_39824257, and Chr03_39824268 with PRI.L resistance (**Table 1**). Among these SNPs, Chr03_37616128 was associated with both PRI19 and PRI.L resistance; Chr03_38912965 with both PRI21and PRI resistance; Chr03_39009342 with both PRI21 and PRI resistance; and Chr03_39824257 and Chr03_39824268 with PRI19, PRI, and PRI.L resistance (**Table 1**), indicating that these SNP markers had stable resistance. These SNP markers had an LOD > 4.5 in the *t*-test for associated ASB resistance.

**Table 1:** SNP markers associated with five ashy stem blight pathogen combinations based on six models, listing the closest genes within 2 kb distance.

SNP	Chr	Position (bp)	-Log (P-value) in Tassel			-Log (P-value) using GAPIT 3			Average LOD	Associated pathogen	Gene	Distance between SNP andgene	*t*-test	Beneficial allele related to resistance/BAT477	Unbeneficial allele associated with susceptibility/NY6020-4
			SMR	GLM	MLM	Blink	GLM	MLM					-Log(P-value)		
Chr03_37381665	3	37381665	4.59	5.90	4.19	8.80	6.12	5.61	5.87	PRI19	Phvul.003G157500	<1 kb	6.19	C	T
Chr03_37616128	3	37616128	4.91	5.80	4.19	1.06	5.70	5.28	4.49				6.07	T	C
Chr03_39824257	3	39824257	6.21	7.23	4.64	1.23	6.08	5.65	5.17		Phvul.003G175900	on gene	6.43	A	G
Chr03_39824268	3	39824268	5.85	6.66	4.36	1.14	5.88	5.48	4.89				6.33	A	G
Chr03_38912965	3	38912965	4.28	4.61	2.83	3.70	4.23	3.39	3.84	PRI21			5.07	G	A
Chr03_38926573	3	38926573	3.13	3.54	2.50	3.43	3.96	3.30	3.31				4.54	T	C
Chr03_39009342	3	39009342	4.41	5.07	2.60	4.19	4.71	3.65	4.10		Phvul.003G168800	< 2 kb	5.25	G	A
Chr03_35546329	3	35546329	3.78	4.83	2.61	3.87	5.12	3.64	3.97	PRL19			5.29	G	C
Chr03_35847673	3	35847673	4.86	5.65	3.11	3.49	4.70	3.51	4.22		Phvul.003G148000	< 1 kb	5.63	G	A
Chr03_36036641	3	36036641	4.53	5.49	2.95	3.71	4.95	3.67	4.21				5.71	G	A
Chr03_36036679	3	36036679	4.51	5.32	2.85	3.68	4.91	3.66	4.15				5.72	C	A
Chr03_38912965	3	38912965	6.49	6.98	3.86	0.42	5.70	4.34	4.63	PRI			6.93	G	A
Chr03_39009342	3	39009342	5.85	7.05	3.51	9.29	6.41	4.83	6.16		Phvul.003G168800	< 2 kb	6.90	G	A
Chr03_39824257	3	39824257	5.38	6.62	3.82	0.65	6.14	5.10	4.62		Phvul.003G175900	on gene	6.63	A	G
Chr03_39824268	3	39824268	5.42	6.44	4.03	0.64	6.12	5.19	4.64				6.67	A	G
Chr03_37616128	3	37616128	5.41	6.76	4.01	0.82	6.40	4.91	4.72	PRI.L			6.84	T	C
Chr03_38912965	3	38912965	6.53	7.17	3.66	0.34	5.82	4.08	4.60				6.83	G	A
Chr03_39009342	3	39009342	6.03	7.46	3.59	9.73	6.70	4.79	6.38		Phvul.003G168800	< 2 kb	7.05	G	A
Chr03_39824257	3	39824257	5.80	7.27	3.88	0.64	6.36	4.96	4.82		Phvul.003G175900	on gene	6.61	A	G
Chr03_39824268	3	39824268	5.72	6.96	3.83	0.55	6.27	4.94	4.71				6.59	A	G

The closest gene for Chr03_37381665 was Phvul.003G157500 with < 1 kb distance; for both Chr03_39824257 and Chr03_39824268 the gene Phvul.003G175900; Chr03_39009342 close to Phvul.003G168800 with a < 2 kb distance; and Chr03_35847673 to Phvul.003G148000 with <1 kb (**Table S4**), indicating that these genes may be associated with ASB resistance.

### Genetic mapping and QTL analysis

Eleven genetic maps consisting of of 35,787 SNPs from Pv01 to Pv11 were built by MSTmap (http://mstmap.org/) and JoinMap 4. There were 3,952 SNPs on Pv01; 3,841 SNPs on Pv02; 7,746 SNPs on Pv03; 2,366 SNPs on Pv04; 4,514 SNPs on Pv05; 2,358 SNPs on Pv06; 1,225 SNPs on Pv07; 3,712 SNPs on Pv08; 1,512 SNPs on Pv09; 2,815 SNPs on Pv10; and 1,746 SNPs on Pv11. The order of SNPs on each genetic map on Pv01, Pv03, Pv04, Pv07, Pv08, and Pv09 match well with their physical maps; Pv02 matches but not for the region from 12 Mbp to 26 Mbp; Pv05 and Pv10 had many SNPs located at the centromere and did not match well; Pv11 had a gap near the centromere; and Pv06 did not match well except from 25 Mbp up (**Figure S8**). This indicates that we can do QTL mapping for ASB resistance on Pv01, Pv03, Pv04, Pv07, Pv08, and Pv09; and it may be possible on Pv11 and partial regions of other chromosomes based on the 126 RIL derived from BAT 477 and NY6020-4.

**Figure 4:**
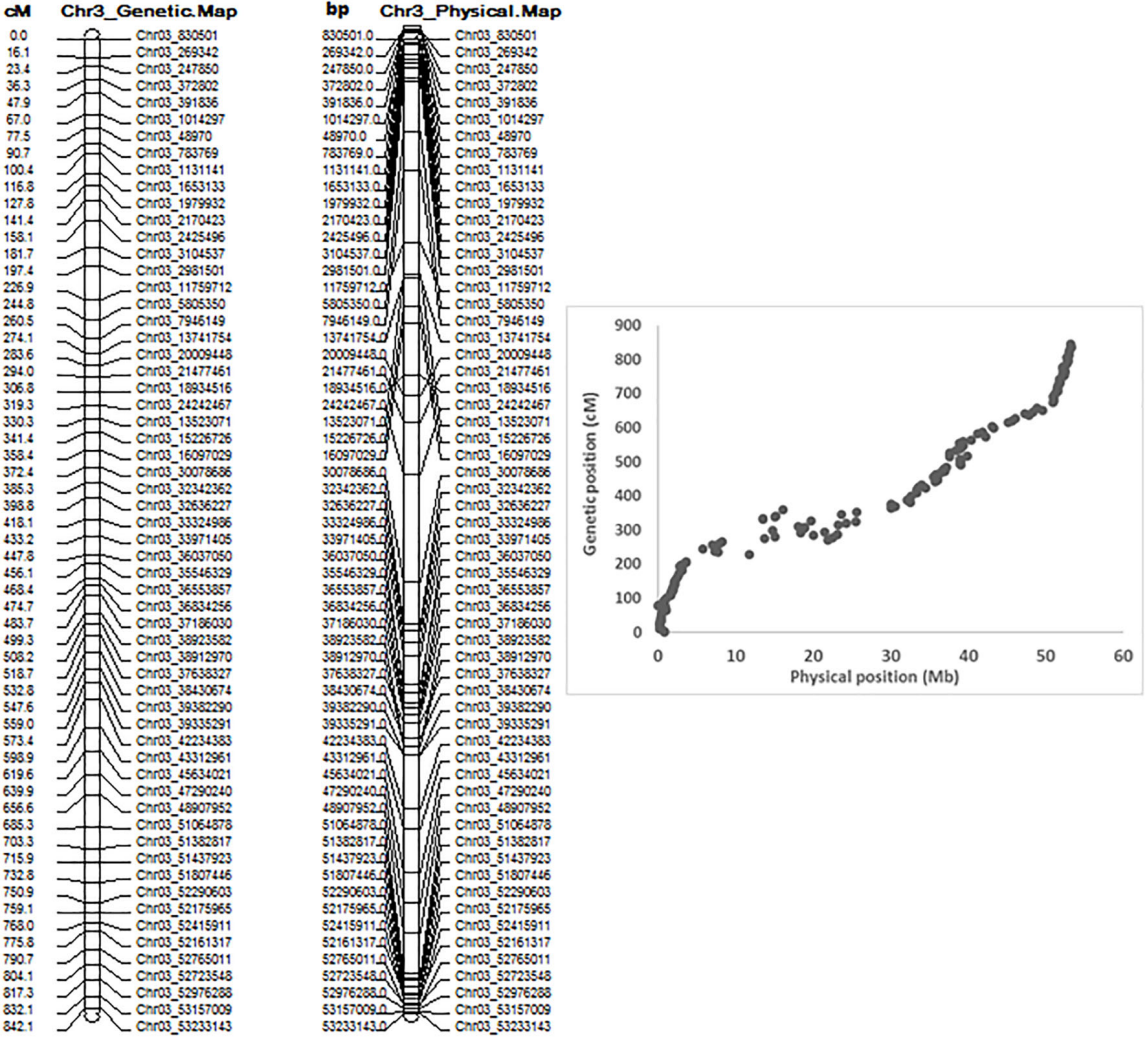
Genetic map (left), physical map, and combined map of physical and genetic map (right) consisting of 179 SNPs on chromosome 3 from 126 common bean RIL of BAT 477 and NY6020-4.

QTL mapping by QGene showed that ASB resistance was observed only on chromosome Pv03. The 7,746 SNPs of Pv03 were too dense to do QTL mapping with a small RIL population with 126 individuals, and so we selected 179 SNPs on Pv03 to create a new linkage map to do QTL analysis for ASB resistance. The genetic and physical positions of the two linkage maps consisted of either 7,746 SNPs and 179 SNPs, listed in **Table S5**, where both combined maps between physical distance (Mbp) and genetic position (cM) were also included. The genetic map of Pv03 matches well to its physical map based on 179 SNPs (**Figure 4**).

**Figure 5:**
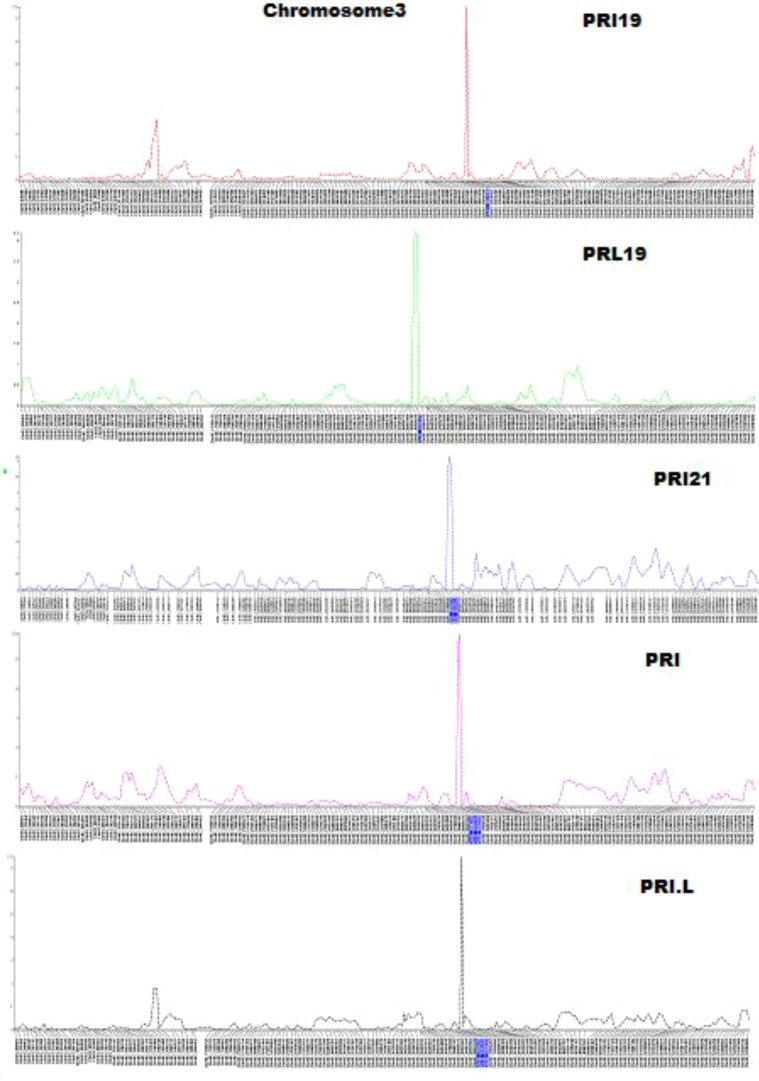
Five genetic maps at the QTL region on chromosome 3 created by single-trait multiple interval mapping (SMIM), where x-axis presents genetic map with SNP markers and y-axis presents LOD value (The detail information including the marker names was shown in **Supplementary Figure S9**).

**Figure 6:**
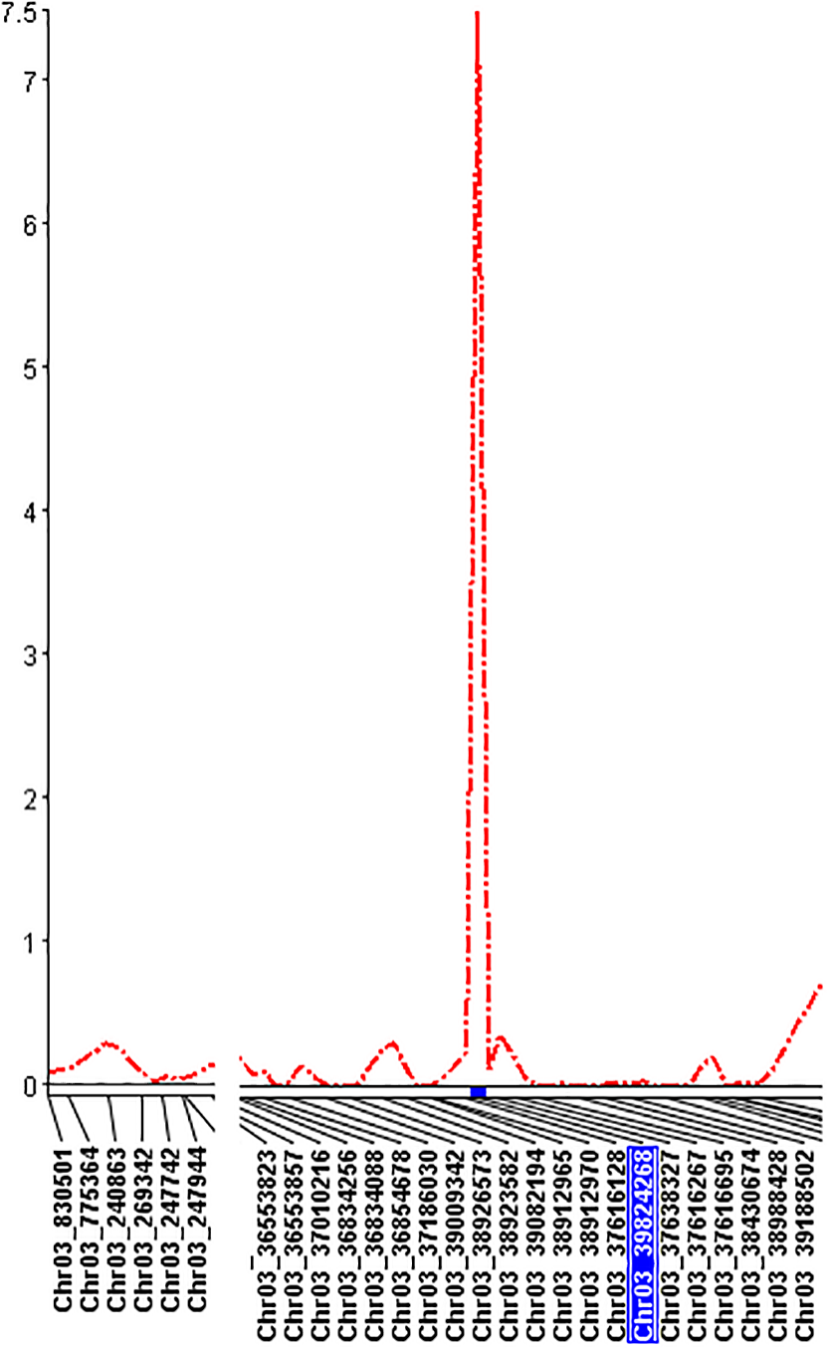
The genetic map at the QTL region with the viewable SNPs on chromosome 3 created by single-trait multiple interval mapping (SMIM) for PRI19 *M. phaseolina* isolate resistance, where the x axis indicates genetic map with SNP markers and the y axis indicates LOD value.

QTL mapping by single-trait multiple interval mapping (SMIM) in Qgene showed a peak on chromosome Pv03 for each of PRI19, PRL19, PRI21, PRI, and PRI.L resistance (**Figure 5**) and the detailed QTL mapping in Pv3 for each ASB resistance was showed in the **Supplementary Figure S9** with viewable and readable SNP marker names. The detailed QTL regions are shown in **Supplementary Figure 10** in order to see the linked SNP markers, and an example of QTL mapping for PRI19 resistance included in the test can be found in **Figure 6**.

Twenty SNPs located at 446.5 - 555.9 cM on Pv03 were linked to ASB resistance in one of five combinations, either PRI19, PRL19, PRI21, PRI, or PRI.L, based on SMR model in QGene (**Table S6**). QTL was identified at 452 – 514 cM on Pv03 based on SMIM model and at 448 – 554 cM on Pv03 based on SMLE (single-trait CIM MLE, single-trait composite interval mapping maximum likelihood estimation) for the five ASB combinations (**Table S6**), indicating that there is a QTL in the region for ASB resistance.

For PRI19 resistance, the QTL peak is at 514 cM of Pv03 based on SMR, SMIM, and SMLE analysis, confirmed by the two SNPs, Chr03_37616128 and Chr03_39824268 (**Tables 2** and **S6**), and closer to Chr03_39824268 based on the peak of SMIM mapping (**Figures 5**, **6**, **S9** and **S10**), and the SNP is on the gene Phvul.003G175900.

**Table 2:** QTL and linked SNP markers for ashy stem blight resistance of five combinations based on three models in Qgene.

Mapping model	SNP	Position /region (cM)	Add effect	LOD	%R^2^
Single marker regression (SMR)	Chr03_37616128	513.5	-0.456	4.718	15.8
	Chr03_39824268	515.7	-0.472	5.032	16.8
Single-trait multiple interval mapping (SMIM)	Chr03_37616128 - Chr03_39824268	514	-0.565	7.488	23.9
Single-trait CIM MLE (SMLE)		514	-0.474	5.141	17.1
Single marker regression (SMR)	Chr03_36036679	451.5	-0.421	4.503	15.2
	Chr03_35546329	456.1	-0.399	4.002	13.6
Single-trait multiple interval mapping (SMIM)	Chr03_36036679 - Chr03_35546329	452	-0.402	3.967	13.5
		454	-0.419	4.191	14.2
		456	-0.401	4.115	14
Single-trait CIM MLE (SMLE)		452	-0.402	3.967	13.5
		454	-0.419	4.191	14.2
		456	-0.401	4.115	14
Single marker regression (SMR)	Chr03_39009342	489.9	-0.477	3.903	13.3
	Chr03_38926573	494	-0.447	3.574	12.2
Single-trait multiple interval mapping (SM IM)	Chr03_39009342- Chr03_38926573	490	-0.459	3.752	12.8
		492	-0.495	4.11	13.9
Single-trait CIM MLE (SMLE)		490	-0.459	3.752	12.8
		492	-0.495	4.11	13.9
Single marker regression (SMR)	Chr03_39082194	503.4	-0.412	4.622	15.5
	Chr03_38912965	507.9	-0.452	5.726	18.9
	Chr03_38912970	508.1	-0.448	5.585	18.5
	Chr03_37616128	513.5	-0.432	5.127	17.1
	Chr03_39824268	515.7	-0.457	5.766	19
Single-trait multiple interval mapping (SMIM)	Chr03_39082194- Chr03_39824268	504	-0.453	5.403	17.9
		506	-0.469	5.857	19.3
		514	-0.202	0.483	1.7
Single-trait CIM MLE (SMLE)		504	-0.453	5.403	17.9
		506	-0.469	5.857	19.3
		508	-0.436	5.308	17.6
		510	-0.46	5.528	18.3
		512	-0.455	5.418	18
		514	-0.446	5.595	18.5
Single marker regression (SMR)	Chr03_37616128	513.5	-0.418	5.506	18.2
	Chr03_39824268	515.7	-0.43	5.823	19.2
Single-trait multiple interval mapping (SMIM)	Chr03_37616128- Chr03_39824268	514	-0.491	7.481	23.9
Single-trait CIM MLE (SMLE)		514	-0.428	5.921	19.5

For PRL19 resistance, the QTL peak is at 451 - 456 cM of Pv03 based on SMR, SMIM, and SMLE analysis, confirmed by the two SNPs, Chr03_36036679 and Chr03_35546329 (**Tables 1** and **S6**; **Figures 5**, **6**, **S9** and **S10**), and a dozen genes are located at the region.

For PRI21 resistance, the QTL peak is at 490 - 494 cM of Pv03 based on SMR, SMIM, and SMLE analysis, confirmed by the two SNPs, Chr03_39009342 and Chr03_38926573 (**Tables 2** and **S6**; **Figures 5**, **6**, **S9**, **S10**), and three genes, Phvul.003G168500, Phvul.003G168700, and Phvul.003G168800 are located at this region.

For PRI resistance, the QTL peak is at 504 - 514 cM of Pv03 based on SMR, SMIM, and SMLE analysis, confirmed by the two SNPs, Chr03_39082194 and Chr03_39824268 (**Tables 2** and **S6**), and closer to Chr03_38912965 based on the peak of SMIM mapping (**Figures 5**, **6**, **S9**, **S10**), and dozens genes are located at this region.

For PRI.L resistance, the QTL peak is at 514 cM of Pv03 based on SMR, SMIM, and SMLE analysis, confirmed by the two SNPs, Chr03_37616128 and Chr03_39824268 (**Tables 2**, **S6**), and closer to Chr03_39824268 based on the peak of SMIM mapping (**Figures 5**, **6**, **S9**, **S10**), and the SNP is on the gene Phvul.003G175900, which showed similar PRI19 resistance.

### Candidate gene identification/detection

There are 305 genes in the QTL region from 36.17 Mbp to 9.83 Mbp on chromosome Pv03 for ASB to PRI19, PRI21, PRL19, PRI, and PRI.L, based on six GWAS models in GAPIT 3 and three QTL models in QGene (**Table S7**). Among the 305 genes, there are 11 disease gene analogues (**Table 3**), where three genes, Phvul.003G152900, Phvul.003G156366, and Phvul.003G168000, link to one or more SNP markers identified by GWAS and listed in **Table S4**. Four genes, Phvul.003G148000, Phvul.003G157500, Phvul.003G168800, and Phvul.003G175900, are located at an associated SNP marker for ASB with < 2kb distance (**Table 3**) based on the GWAS and QTL analyses in **Tables 1** and **2**.

**Table 3:** Eleven disease gene analogues located at the QTL region between 35.8 Mbp and 39.9 Mbp on chromosome Pv03, and four genes located within 2 Kb distance from one or more SNP associated with ashy stem blight resistance.

Gene	Chr	Gene_Start_pos	Gene_End_pos	Gene-defined	Close SNP	From gene start	From gene end	Comment	
Phvul.003G152900	3	36779067	36784453	Leucine-rich repeat protein kinase family protein	Chr03_36834088	55021	49635	< 50 kb	SNP markers listed within 50 Kb distance
					Chr03_36834256	55189	49803	< 50 kb	
Phvul.003G154000	3	36948002	36951212	Leucine-rich receptor-like protein kinase family protein					
Phvul.003G156366	3	37209311	37212260	Leucine-rich repeat protein kinase family protein	Chr03_37185993	-23318	-26267	< 25 Kb	
					Chr03_37186030	-23281	-26230	< 25 Kb	
					Chr03_37186035	-23276	-26225	< 25 Kb	
Phvul.003G158700	3	37486636	37489949	Cysteine-rich RLK (receptor-like protein kinase)					
Phvul.003G159700	3	37734035	37737603	Leucine-rich repeat protein kinase family protein					
Phvul.003G161500	3	38073321	38075049	Protein kinase superfamily protein					
Phvul.003G163700	3	38264295	38267316	P-loop containing nucleoside triphosphate hydrolases superfamily protein					
Phvul.003G165700	3	38535457	38548714	Protein kinase superfamily protein					
Phvul.003G168000	3	38867946	38872171	Protein kinase superfamily protein	Chr03_38912965	45019	40794	< 45 kb	
					Chr03_38912970	45024	40799	< 45 kb	
Phvul.003G170900	3	39293276	39299144	Avirulence induced gene (AIG1) family protein					
Phvul.003G172400	3	39452306	39462226	Leucine-rich repeat family protein					
Phvul.003G148000	3	35848190	35865660	FGGY family of carbohydrate kinase	Chr03_35847673	-517	-17987	< 1kb	< 2 Kb
Phvul.003G157500	3	37378080	37381316	Tetratricopeptide repeat (TPR)-like superfamily protein	Chr03_37381665	3585	349	< 1kb	
Phvul.003G168800	3	39004746	39007688	Raffinose synthase family protein	Chr03_39009342	4596	1654	< 2 kb	
Phvul.003G175900	3	39822021	39824655	Drought sensitive, WD repeat-containing protein 76	Chr03_39824257	2236	-398	on gene	
					Chr03_39824268	2247	-387	one gene	

## Discussion

### Ashy stem blight resistance in the RIL

In this study, BAT 477 showed intermediate to high ABS resistance, and NY6020-4 was intermediately susceptible to ABS based on PRI19 and PRL19 *M. phaseolina* isolates collected from common bean fields planted in Isabela and Lajas, Puerto Rico, respectively, in October 2019; PRI21 isolate collected from an infected seedling planted in the greenhouse in Isabela in January 2021; PRI (combined PRI19 and PRI21); and PRI.L (combined PRI19, PRL19, and PRI21) (**Table S8**). Although the ASB rate difference between the two parents was not large, the 126 RIL showed large variation, with an extensive range for PRI19, PRL19, PRI21, PRI, and PRI.L between the two parents (**Figure 1**, **Table S1**), confirming the suitability of the RIL for GWAS and QTL analyses. High broad-sense heritability (46.3% - 71.2%) was also observed (**Table S2**), indicating that ASB resistance in BAT 477 can be transferred to other common bean cultivars and lines.

### Variability of *Macrophomina phaseolina*

In this study, three *M. phaseolina* pathogen sources, PRI19, PRI21, and PRL19, were used to evaluate ASB resistance in the RIL. Although we were unsure whether they belonged to the same race, similar results were observed with variability (**Figure 1**; **Tables S1, S2**), and strong correlations (r = 0.36 - 0.98) also observed with majority (80%) r > 0.60 (**Table S3**). QTL and association mapping of ASB resistance showed the same QTL region on chromosome 3 for ASB resistance for PRI19, PRL19, PRI21, PRI, and PRI.L, but different significant SNP markers for each pathogen source were identified (**Tables 1**, **2**; **Tables S4, S6**; **Figures 6**, **S9**, **S10** and **S11**), indicating that there was variability of the *M. phaseolina* pathogen used in this study. The variability of the *M. phaseolina* pathogen was reported by Reyes-Franco et al. (2006); Mahdizadeh et al. (2012), and Yesil and Bastas (2016), who also studied the genetic diversity of *M. phaseolina* collected from Iran, Mexico, Turkey, and other countries.

### QTL identification of ashy stem blight resistance

QTL mapping is based on phenotypic data and genotypic data (molecular markers) to map QTL to chromosome(s) or linkage group(s) (LGs) in segregating population(s) such as F_2_, F_2:3_, or RIL using a statistic model, and it has been widely used in tagging major or minor genes/alleles in crops. Except for single marker analysis such as single marker regression and *t*-test, QTL mapping requires an LG or chromosome with ordered markers, known as genetic maps. Different genetic maps will result in different results for QTL mapping. The marker number, marker density, and marker order in each chromosome or LG affect the results in QTL mapping, as do the mapping populations. Even using same marker number, the marker order in each chromosome or LG will be different depending on the mapping populations (parents, generation, size, etc.) and mapping tools such as MSTmap and JoinMap.

In this study, we used JoinMap 4 (Van Ooijen, 2006) and MSTmap (Wu et al., 2008; http://mstmap.org/) to create the genetic linkage maps in an RIL population of 126 F_6:7_ for RIL derived from a cross between BAT 477 and NY6020-4. We found it was easy to create genetic maps but hard to create stable and uniform genetic maps of the 11 chromosomes. The order of the SNPs in each chromosome was different depending on the SNP number, but the physical position of the SNPs did match well on the chromosomes. Although a total of 6,463,014 SNPs were identified in the 126 RIL and their parents, distributed on the 11 chromosomes, and 35,787 SNPs mapped to create the genetic maps (**Figure S8**), the genetic and physical distances and the order of SNPs in each chromosome still did not match well. However, the chromosome Pv03 did have good matched genetic and physical maps, using either 7,746 SNPs or 179 SNPs (**Table S5**, **Figure 4**), on which we identified the QTL for ASB resistance. The orders of the genetic and physical maps in the QTL region were not exactly the same (**Table S5**), such as for the three ABS SNP markers, Chr03_39009342, Chr03_37616128, and Chr03_39824268, where the physical order was Chr03_37616128-Chr03_39009342-Chr03_39824268 with position 39,009,342 bp, 37,616,128 bp, and 39,824,268 bp, respectively, on Pv03, but their genetic map order was Chr03_39009342-Chr03_37616128-Chr03_39824268 with genetic position 489.999 cM, 513.509 cM, and 515.756 cM, respectively, on Pv03, based on 179 SNPs on this chromosome; however, the genetic order was Chr03_39009342-Chr03_39824268- Chr03_37616128 based on 7,746 SNPs on chromosome Pv03 (**Tables 2**, **S5**), which may be caused by the map population size with 126 RIL.

In order to overcome the disadvantage of QTL mapping caused by the genetic order error, we also performed GWAS for ASB resistance in this RIL using three models – GLM, MLM, and Blink – in GAPIT 3 and three models – SMR, GLM, and MLM – in TASSEL 5 when PCA = 2, and combined QTL mapping using SMR, SMIM, and SMLE in QGene. A QTL was identified to be located at 35,546,329 - 39,826,434 bp on Pv03, and two SNPs, Chr03_39824257 and Chr03_39824268, located at 39,824,257 bp and 39,824,268 bp on Pv03, respectively, were identified as being the strongest markers associated with ASB resistance in this study.

Resistant QTL to ASB derived from the BAT 477 breeding line have been reported in previous studies (Mayek-Pérez et al, 2009; Méndez-Aguilar et al., 2017) with different results. Mayek-Pérez et al. (2009) reported that BAT 477 had two and nine genes for *M. phaseolina* resistance in field conditions. Hernández-Delgado et al. (2009) detected one QTL associated to charcoal rot resistance in BAT 477 using a F2 population and the markers BPC40M127 and BPC54M150 associated with charcoal rot (=ASB) resistance (Méndez-Aguilar et al., 2017). Méndez-Aguilar et al. (2017) identified QTL for ABS resistance in a 94 F_2:9_ RIL population derived from a cross between BAT 477 and cv. Pinto UI-114 using 476 AFLP polymorphic markers, and mapped the QTL on Pv03, Pv05, Pv06, Pv08, Pv09, and Pv10 LG based on 68 AFLP markers distributed in 10 linkage groups (LG) with coverage of 718.1 cM and two QTL in Pv03 by use of only six AFLP markers on Pv03. The ASB resistant QTL on Pv03 was identified using 7,746 SNPs on chromosome 3 by QTL and associated mapping with several models.

However, these reported QTL were identified under natural infestations of *M. phaseolina* in the field, where avoidance mechanisms (i.e., plants with upright growth habits, open canopy, and/or resistance to lodging) may be associated with lower severity to this pathogen (Mayek-Pérez et al., 2001b; Viteri and Linares, 2022a). However, our novel QTL on Pv03 chromosome was identified in the greenhouse, which is the appropriate environment used to detect physiological resistance to necrotrophic fungus such as *M. phaseolina* (Viteri and Linares, 2017; Viteri et al., 2019) and *Sclerotinia sclerotiorum* L. de Bary (Soule et al., 2011; Schwartz and Singh, 2013; Viteri et al., 2015).

### Candidate gene for ashy stem blight resistance

There are 305 genes in the QTL region from 36.17 Mbp to 9.83 Mbp on chromosome Pv03 for ASB resistance to PRI19, PRI21, PRL19, PRI, and PRI.L based on six GWAS models in GAPIT 3 and three QTL models in QGene (**Table S7**). Among the 305 genes, there are 11 disease gene analogues (**Table 3**), which may be associated with the ASB resistance. From this study, the QTL for ASB resistance in the RIL of BAT 477/NY6020-4 was located at 35,546,329 - 39,826,434 bp on Pv03. Two SNPs, Chr03_39824257 and Chr03_39824268 located at 39,824,257 bp and 39,824,268 bp on Pv03, respectively, were identified as the strongest markers associated with ASB resistance, and they were on the gene Phvul.003G175900 (drought sensitive, WD repeat-containing protein 76), thus Phvul.003G175900 located at 39,822,021 – 39,824,655 bp on Pv03 was recognized as the candidate for ASB resistance in the RIL. The two SNP markers and the gene can provide information for selecting ASB resistance in common bean breeding through MAS.

### Utilization of the RILs for ashy stem blight resistance

Among 126 RIL, 10 lines showed high resistance to ASB pathogens, with 4 or lower as an average score across two years in two locations (**Supplementary Table S8**), where either PRI.L or RPI score was <= 4; PRI19 or PRI19 <= 3.8 (except 20373Vit_92 with score = 4.1); and PRI21 <=4.1 (except 20373Vit_85 with score = 4.8 and 20373Vit_128 = 4.2), indicating that the 10 RIL were more ASB resistant in this RIL population, suggesting they can be used as parents in common bean breeding.

The 126 RIL can be divided into two clusters (groups) (**Figure S11**) based on each of the two parents, BAT 477 and NY 6020-4. The top 10 ASB resistant RIL were also distributed into two groups analyzed by MEGA 7 using Maximum Likelihood (ML) method either among 128 lines (126 RILs plus 2 parents) or 12 lines (10 R-line plus two parents) (**Figure S11**), indicating that the ASB resistant QTL ‘ASB-qtl-3’ on chromosome Pv03 can be transferred from the BAT 477 breeding line to an NY 6020-4 genetic background and utilized in common bean breeding programs to develop new ASB resistant lines. New common bean germplasms UPR-Mp-42 and UPR-Mp-48 have been developed with BAT 477, Andean PRA154, and NY6020-4 as parents in their lineage, with enhanced levels of resistance to ASB (Viteri and Linares, 2022b). However, it has been necessary to pyramid higher levels of resistance derived from the Andean gene pool (i.e., A 195, ‘PC 50’, and PRA154) (Viteri and Linares, 2017; Viteri et al., 2019). It has been reported that BAT 477 and NY6020-4 can reach susceptible scores under a severe screening method (i.e., two inoculations per plant) (Viteri and Linares, 2022b).

## Conclusion

In this study, a QTL region for ASB resistance was identified in an RIL population derived from BAT 477 and NY6020-4. The QTL was located at 35,546,329 - 39,826,434 bp on chromosome Pv03. Two SNPs, Chr03_39824257 and Chr03_39824268 located at 39,824,257 bp and 39,824,268 bp on Pv03, respectively, were identified as the strongest markers associated with ASB resistance, and they were on the gene Phvul.003G175900 (drought sensitive, WD repeat-containing protein 76), thus Phvul.003G175900 located at 39,822,021 – 39,824,655 bp on Pv03 was recognized as the candidate for ASB resistance in the RIL. The two SNP markers and the gene can provide information for selecting ASB resistance in common bean breeding through MAS.

## Data availability statement

The datasets presented in this study can be found in online repositories. The names of the repository/repositories in FigShare https://doi.org/10.6084/m9.figshare.19919221.v1 and accession number(s) can be found in the article/**Supplementary Material**.

## Author contributions

DV was the principal investigator (PI) for the project. DV, AL, and ZM were involved in phenotyping and performing ashy stem blight resistant evaluation. AS performed genomic and statistical analysis. AS and DV wrote the draft of the manuscript. All authors contributed to the article and approved the submitted version.

## Acknowledgments

We thank the USDA-NIFA regional project S-009 (Award number: 1017544) for providing the funds for this research. The authors also thank the Texas A&M AgriLife Genomics and Bioinformatics Service for the sequencing and basic bioinformatics analysis to provide raw DNA sequencing and SNP data. In addition, we thank Luis Cabán and Arturo Luciano for their support in the field and harvesting activities.

## Conflict of interest

The authors declare that the research was conducted in the absence of any commercial or financial relationships that could be construed as a potential conflict of interest.

## Publisher’s note

All claims expressed in this article are solely those of the authors and do not necessarily represent those of their affiliated organizations, or those of the publisher, the editors and the reviewers. Any product that may be evaluated in this article, or claim that may be made by its manufacturer, is not guaranteed or endorsed by the publisher.
